# Vitamin D: Nutrient, Hormone, and Immunomodulator

**DOI:** 10.3390/nu10111656

**Published:** 2018-11-03

**Authors:** Francesca Sassi, Cristina Tamone, Patrizia D’Amelio

**Affiliations:** Department of Medical Science, Gerontology and Bone Metabolic Diseases, University of Turin, 10126 Turin, Italy; francesca.sassi@unito.it (F.S.); cristinatamone78@gmail.com (C.T.)

**Keywords:** vitamin D, immune system, gut microbiota, autoimmune diseases, T cells

## Abstract

The classical functions of vitamin D are to regulate calcium-phosphorus homeostasis and control bone metabolism. However, vitamin D deficiency has been reported in several chronic conditions associated with increased inflammation and deregulation of the immune system, such as diabetes, asthma, and rheumatoid arthritis. These observations, together with experimental studies, suggest a critical role for vitamin D in the modulation of immune function. This leads to the hypothesis of a disease-specific alteration of vitamin D metabolism and reinforces the role of vitamin D in maintaining a healthy immune system. Two key observations validate this important non-classical action of vitamin D: first, vitamin D receptor (VDR) is expressed by the majority of immune cells, including B and T lymphocytes, monocytes, macrophages, and dendritic cells; second, there is an active vitamin D metabolism by immune cells that is able to locally convert 25(OH)D_3_ into 1,25(OH)_2_D_3_, its active form. Vitamin D and VDR signaling together have a suppressive role on autoimmunity and an anti-inflammatory effect, promoting dendritic cell and regulatory T-cell differentiation and reducing T helper Th 17 cell response and inflammatory cytokines secretion. This review summarizes experimental data and clinical observations on the potential immunomodulating properties of vitamin D.

## 1. Introduction

The role of vitamin D in the regulation of calcium-phosphate homeostasis and in the control of bone turnover is well known. Vitamin D status significantly affects skeletal health during growth and in adult age, its deficiency during growth leads to rickets [1], whereas during adult age it is responsible of osteomalacia and various degree of osteoporo-malacia [2]. Low vitamin D status increases bone turnover, decreases bone density, and is associated with increased fracture risk. In addition to the well-known effect on skeletal health in the last two decades evidence has been accumulated on the pleiotropic effect of vitamin D other than on bone health thanks to the findings that vitamin D receptor (VDR) and the vitamin D activating enzyme 1-α-hydroxylase (CYP27B1) are expressed in several cells outside the bone and kidney, such as in the intestine, platelets, pancreas, and prostate [3]. Several cells involved in the immune function express VDR and CYP27B1, this observation suggests that the active form of vitamin D, 1,25(OH)_2_D_3_, is able to control the immune function at different levels. Previous reviews on the role of vitamin D in the regulation of immune system have been published in recent years [4,5]. Here we summarize the recent evidence sexploiting authors’ expertise in both experimental and clinical fields.

## 2. Vitamin D Metabolism

Vitamin D enters the body trough dietary intake (about 20% of vitamin D_3_ is assumed with diet) or is synthetized by the skin (80%) from 7-dihydrocholesterol following UVB exposure. Vitamin D_3_ becomes biologically active after hydroxylation in the liver by the enzymes cytochrome P450 2R1 (CYP2R1) and cytochrome P450 27 (CYP27A1) becoming 25(OH)D_3_. The fully-active metabolite 1,25(OH)_2_D_3_ is hydroxylated in the kidney by the enzyme CYP27B1, parathormone (PTH), and the fibroblast growth factor 23 (FGF-23) control CYP27B1 synthesis and activity [6]. Synthesis of 1,25(OH)_2_D_3_ is strictly regulated in a renal negative feedback loop: high levels of 1,25(OH)_2_D_3_ and FGF-23 inhibit CYP27B1 and induce the cytochrome P45024A1(CYP24A1), which transforms 1,25(OH)_2_D_3_ into the inactive form 24(OH)D_3_ [7].

In addition to the kidney, CYP27B1 is expressed by other cell types, including immune cells. These cells produce 1,25(OH)_2_D_3_ that has autocrine and/or paracrine effects, the high level produced locally is thought to be responsible for immunomodulation. The regulation of CYP27B1 synthesis in immune cells is different than the signals regulating kidney production of 1,25(OH)_2_D_3_. Inflammatory signals, such as lipopolysaccharide (LPS) and cytokines, induce monocyte and macrophage production of CYP27B1 [8,9,10]. These differences in the regulation of 1,25(OH)_2_D_3_ production point to an autocrine/paracrine effect as immunomodulatory.

## 3. Vitamin D Status

Vitamin D status is defined by the blood measurement of its hydroxylated form 25(OH)D_3_, however, there is no common agreement on the threshold levels to identify desirable vitamin D level. Guidelines from different scientific societies and different countries established 50 nM/L or 75 nM/L to consider vitamin D sufficiency [11,12,13], however, it is generally accepted that 25(OH)D_3_ levels lower than 50 nM/L are associated with bone metabolism alteration, increased risk of falls, and myopathy in adults [14,15,16,17,18]. Experts in the field generally agree to maintain 25(OH)D_3_ between 20 and 125 nM/L in order to obtain the certain skeletal effects without toxic effects. Recent literature raises the suspicion that administration of a bolus of vitamin D_3_ higher than 50,000 UI may result in an increased risk of falls and fractures [19,20]; moreover, the mortality related to 25(OH)D_3_ is a “U shaped curve” and 25(OH)D_3_ levels higher than 150 nM/L are associated with increased mortality [21].

## 4. Vitamin D and the Innate Immune System: Antimicrobial Activity

The innate immune system is the first defense against infection, it is required to rapidly fight against invading pathogens. The innate immune system comprehends components both from the host and resident microbes (microbiota). The host defense comprises physical barriers to infection (as skin, mucous surfaces, mucus, and vascular endothelial cells), enzymes expressed by epithelial and phagocytic cells (as lysozyme), antimicrobial peptides and proteins (as defensins, cathelicidins, and others expressed by phagocytes), inflammatory humoral components (as complement and opsonins), and cell receptors that rapidly recognize pathogens (as toll-like receptors) and cellular components (as mast cells, dendritic cells, macrophages, neutrophils cells and natural killer). Interaction between microbiota a vitamin D will be analyzed in the following paragraph. 

Vitamin D is a well-known regulator of innate immunity, the first data on this topic have been generated on the treatment of diseases caused by mycobacteria, such as tuberculosis and leprosy [22,23], however, the mechanisms responsible for these actions have been elucidated in more recent years. 1,25(OH)_2_D_3_ enhances the production of defensin β2 and cathelicidin antimicrobial peptide (CAMP) by macrophage and monocyte keratinocytes increasing their antimicrobial activity [24,25,26]. Moreover, 1,25(OH)_2_D_3_ increases chemotaxis, autophagy, and phagolysosomal fusion of innate immune cells [27,28]. The exposition of human monocytes to pathogens, such as *M. tuberculosis* and others, up-regulates the expression of CYP27B1 and of VDR, thus enhancing both the cell ability to produce 1,25(OH)_2_D_3_ in the site of infection and to respond to this metabolite. However, macrophages are heterogeneous, with different functions [29]. Macrophages formed after interleukin (IL)-15 stimulus respond to vitamin D stimulus increasing their antimicrobial activity, whereas phagocytic macrophages obtained after stimulus with IL-10 are weakly influenced by vitamin D levels regardless oftheir high phagocytic activity [10,30].

1,25(OH)_2_D_3_ up-regulates CAMP not only by monocytes/macrophages, but also in other cells participating in the innate immune system as first-barrier defenses, such as keratinocytes, epithelial, intestinal, lung and corneal cells, and placenta trophoblasts (see for a comprehensive review Wei and Christakos, 2015) [4].

Data in humans on infections other than mycobacterial have been generated on urinary and respiratory infections and on sepsis. A predisposition to urinary tract infection in children with low vitamin D levels due to the reduced production of CAMP and defensing β2 has been suggested by association studies [31,32]. Additionally, in chronic obstructive pulmonary disease (COPD) patients’ levels of CAMP and other antimicrobial peptides were associated with increased risk of acute exacerbations [33]. Consistent with this datum treatment with 1,25(OH)_2_D_3_ was effective in reducing respiratory infections in asthma patients thanks to increased CAMP expression and inflammatory cytokine modulation [34]. Data on the role of vitamin D status and vitamin D supplementation in sepsis are also available both in pediatric and in adult patients: in pediatric patients a clear role for 25(OH)D_3_ and CAMP was not demonstrated [35], whereas in adults lower levels of 25(OH)D_3_ were found in sepsis [36] and a high-dose of vitamin D3 increases circulating CAMP and reduces inflammatory cytokines as IL-6 and IL-1β [37].

More recently data on a possible role of vitamin D in increasing resistance to HIV infection have been published, in particular HIV-exposed seronegative individuals produced more CAMP in oral-mucosa and peripheral-blood, and have higher CYP24A1 mRNA in vaginal-mucosa; CYP24A1 is considered an indicator of high levels of 1,25(OH)_2_D_3_ [38]. Low serum vitamin D has been associated with HIV/AIDS progression and mortality [39].

1,25(OH)_2_D_3_ is able to increase the production of other antimicrobial peptides, such as defensing β2-4, this ability has been demonstrated both in vitro by monocytes stimulation [40,41] and in vivo in pediatric patients’ blood [32].

**Figure 1:**
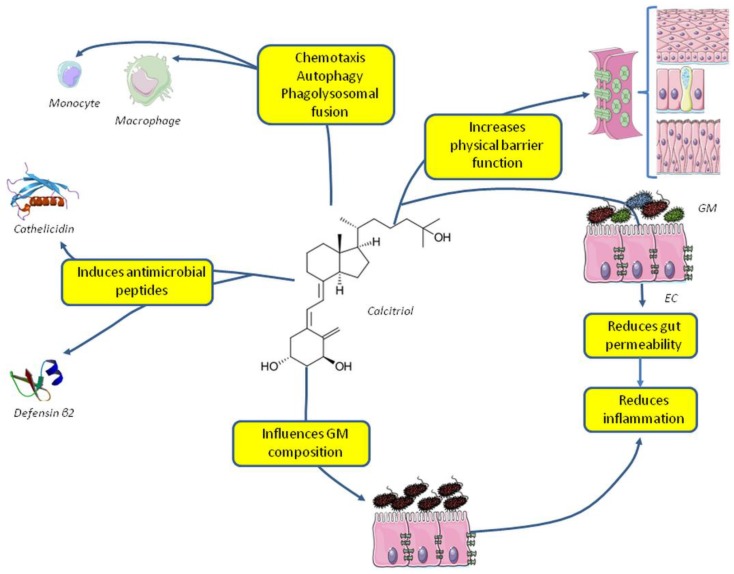
Effects of vitamin D on the innate immune system and gut microbiota. Abbreviations: EC, enteral cells; GM, gut microbiota.

Vitamin D is able to modulate innate immune system, also increasing the phagocytic ability on immune cells [42,43] and by reinforcing the physical barrier function of epithelial cells. In particular 1,25(OH)_2_D_3_can enhance corneal [44] and intestinal [45] epithelial barrier function (Figure 1).

Taken together these data point to a role of vitamin D in defending the organism against pathogens suggesting that vitamin D sufficiency has to be granted in patients affected by acute or chronic infection. The ability of immune cells to hydroxylate 25(OH)D_3_ into its active form 1,25(OH)_2_D_3_ suggests administrating vitamin D_3_ rather than hydroxylated metabolites to patients affected by infections in order to allow the autocrine/paracrine function of 1,25(OH)_2_D_3_ without overcoming local hydroxylation and the feedback system.

## 5. Vitamin D and Microbiota: Increasing Host Defenses

The whole of the commensal, symbiotic, and pathogenic microorganisms living in different areas of the human body has defined microbiota. Microbiota and the host have several relationships, and the perfect balance between microbiota and the host is required for the development, maturation, and properfunction of the immune system [46]. Several papers suggest that vitamin D is one of the actors of the complex relationship between microbiota living in the gut (GM) and immune system modulation. Vitamin D is responsible for the barrier function of the intestinal epithelium and for the modulation of the bowel immune system, hence, low levels may be associated with greater gut permeability and, consequently, with GM-induced metabolic endotoxemia that induces a low-grade inflammation [47]. Moreover, vitamin D administration may influence GM composition, and in vitro data demonstrate that vitamin D enhances macrophages’ ability to kill *Escherichia coli.* [48]. In animals with vitamin D depletion and the knockout of the VDR, the GM dysbiosis favors metabolic disorders [49]. Other studies in mice demonstrated that VDR reduces the response to infection of the intestinal epithelium [50].

Elegant studies in transgenic mice demonstrated that over-expression of VDR in the intestinal epithelium induces resistance to colitis [51,52] and decreases mucosal inflammation suppressing epithelial cell apoptosis, boosting tight junction function [51,53]. On the other hand VDR selective deletion in bowel favors a more severe form of colitis characterized by greater Th1 and Th17 mucosal infiltration and inflammatory cytokines production [54]. In humans, observational studies suggest that low levels of 25(OH)D_3_ are associated with increased risk of inflammatory bowel disease (IBD) [55,56,57] and that high levels of 25(OH)D_3_ in these patients protect against *Clostridium difficile* infection [58]. The experimental data on the role of VDR in developing IBD have been confirmed by the finding of a significant reduction of VDR expression (about 50%) in the colon epithelium in patients affected by IBD with respect to healthy controls [51,53]. The reduction in VDR expression by IBD patients may explain the different effect on GM composition of high oral dosages of vitamin D_3_ demonstrated in a small cohort of patients affected by Crohn’s disease with respect to healthy controls [59], however, human data on the effect of vitamin D supplementation on GM in IBD are still controversial, as other studies did not confirm these results [60,61]. In the study by Luthold and coll. [61] dietary intake of vitamin D and 25(OH)D_3_ were inversely correlated with *Coprococcus* and *Bifidobacterium*, however, thanks to their ability to produce butyrate these bacteria are commonly considered as anti-inflammatory. A possible explanation of these contradictory results may be the different effect of vitamin D on GM according to the different gastro-intestinal tracts considered [62]. Recently, a double-blind placebo-controlled study on patients affected by cystic fibrosis demonstrated that vitamin D insufficiency is associated with different microbiota not only in the gut, but also in the airways, and that the administration of 50,000 IU of oral vitamin D_3_ weekly significantly affects microbiota composition [63]. Nevertheless, the evaluation of clinical outcomes of microbiota change is still open.

Several data point to an effect of vitamin D on microbiota. Conversely, some recent reports suggest that microbiota, per se, influences vitamin D metabolism mainly through FGF-23; germ-free (GF) mice have low vitamin D and high FGF-23, whereas their colonization with bacteria results in increased levels of tumor necrosis factor-α (TNF-α) and a decrease in FGF-23 with normalization of vitamin D hydroxylated metabolites. Inhibition of FGF-23 in GF mice restores vitamin D metabolism without bacterial colonization of the gut [64] (Figure 1).

The role of GM as an active player in the regulation of bone metabolism in humans is being investigated more and more [46], and the role played by vitamin D is still under debate. Further studies to clarify their interplay are needed.

## 6. Vitamin D and the Adaptive Immune System

The adaptive immune system or acquired immune system is the second defense against infection. It is required to specifically fight against pathogens, is activated by exposure to pathogens, and unlike the innate immune system it is able to learn about the pathogen and enhance the immune response accordingly, thanks to an immunological memory. The adaptive immune system is composed of T and B cells and is also responsible for autoimmune reaction. 

25(OH)D_3_ suppresses adaptive immunity [4,65]. In experimental models it down-regulates the immune responses mediated by T helper (Th) 1 cells, thus inhibiting the production of pro-inflammatory cytokines, such as Interferon-γ IFN-γ, IL-6, IL-2, and TNF-α [66,67]. Although experimental studies in vitro and in animals have yielded encouraging results on the immunomodulatory effect of 1,25(OH)_2_D_3_, the same cannot be said about human studies, and few studies have confirmed the suppressive effect of vitamin D on Th1 cells and inflammatory cytokine production in different diseases and spinal tuberculosis [68], uremia [69], and autoimmune thyroiditis [70]; whereas others in IBD [71], dialysis [72], and rheumatoid arthritis [73] do not confirm these results. These discrepancies may be due to the different diseases considered and also to the different type of treatment administered, mainly 1,25(OH)_2_D_3_ in vitro and in animals and vitamin D_3_in vivo in humans. Moreover, when considering administration of vitamin D3 different doses were used in different studies. Therefore, it is almost impossible to compare the results.

It has been suggested that 1,25(OH)_2_D_3_ acts as an immunomodulatory not only by suppressing Th1 cells activation, but also modulating Th2 cells, T regulatory (Tregs) cells activity, and Th17 cells.

The majority of the in vitro studies assessing the effect of vitamin D on Th2 suggests that 1,25(OH)_2_D_3_ upregulates Th2 cells activity [74,75,76]. Amongst immunomodulatory effects of vitamin D its ability to suppress Th17 and increase Treg cells has been recently demonstrated [77,78,79]. Th17 cells produce IL-17 and have been implicated in the pathogenesis ofseveral autoimmune diseases, some experimental studies suggest that 1,25(OH)_2_D_3_ suppresses Th17 formation and activity [67,80,81,82,83] by blocking Nuclear Factor of Activated T-cells (NFAT) and Runt-related Transcription Factor 1 (RUNx1) binding to the IL-17 promoter and inducing Forkhead box P3 (FOXP3) [81], and by inhibiting RAR-related Orphan Receptor Gamma2 (RORγt) which is the transcription factor of IL-17 [84].

More recently our lab showed no effect of the administration of a high bolus of vitamin D_3_ (300,000 UI) in the modulation of Th subset in patients affected by early rheumatoid arthritis [73], as well as a study on hemodialysis patients [72]. 

It has also been suggested that the administration of oral vitamin D_3_ increases Tregs function in patients with type 1 diabetes mellitus [85], however, in other diseases, such as early rheumatoid arthritis, this effect was not confirmed [73].

**Figure 2:**
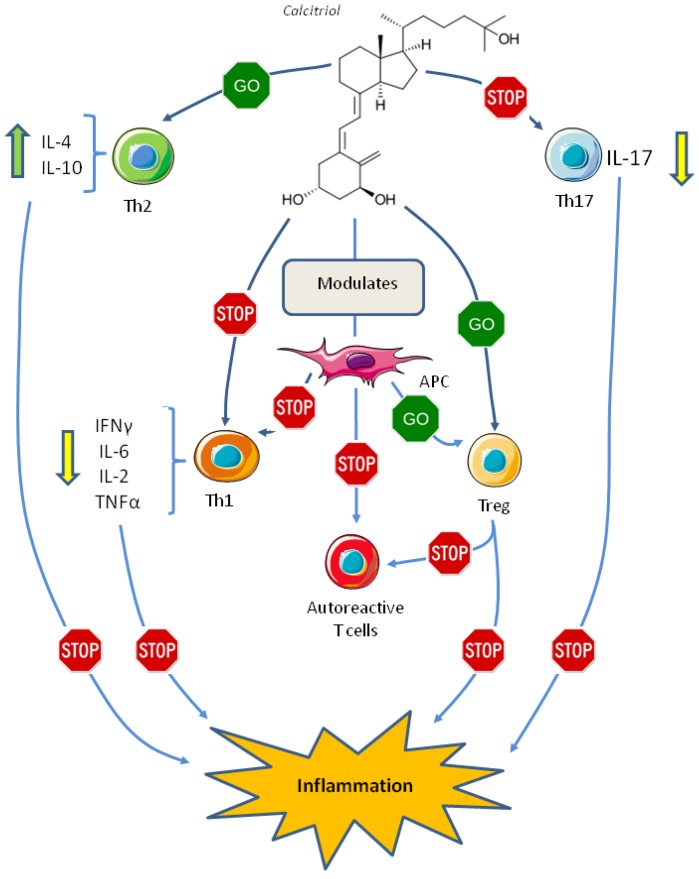
Effect of vitamin D on the adaptive immune system. Abbreviations: APC, antigen presenting cell; IFN, interferon; IL, interleukin; Th1, T helper 1 cell; Th2, T helper 2 cell; Th17, T helper 17 cell; TNF, tumor necrosis factor; Treg, T regulatory cell.

The overall effect of vitamin D on Th cells differentiation may be mediated by its effect on dendritic cells, these cells are antigen-presenting cells (APCs), responsible for T cell differentiation into an effector cell with pro- or anti-inflammatory properties, thus, modulation of APCs is crucial in initiating and maintaining adaptive immune response and self-tolerance [86]. In vitro differentiation of dendritic cells in the presence of 1,25(OH)_2_D_3_ induces a “tolerogenic state” characterized by low levels of inflammatory cytokines, such as IL-12 and TNF-α, with increased levels of the anti-inflammatory IL-10, these cells induce the differentiation of Treg cells and induce apoptosis in the autoreactive T cells [87,88,89,90] (Figure 2).

Taken together these data are not sufficient to prove a real role for vitamin D in the modulation of adaptive immune system in humans, thus, the therapeutic use of vitamin D and its metabolites in patients aiming to ameliorate the adaptive immune system is not sustained by sufficient data.

## 7. Vitamin D and Autoimmune Diseases

Thanks to the evidences of immunomodulatory effect of vitamin D the role of vitamin D deficiency and supplementation in autoimmune diseases has long been studied. Animal studies showed an important role of 1,25(OH)_2_D_3_ supplementation in the control of autoimmune diseases, such as experimental autoimmune encephalomyelitis (EAE) and collagen-induced arthritis (CIA). In these two conditions 1,25(OH)_2_D_3_ prevents the initiation and reduces the disease progression [91,92,93]. Similarly, different mouse models of enterocolitis display a more severe phenotype during vitamin D deficiency and reduced inflammation after administration of 1,25(OH)_2_D_3_ (see for a review Alhassan et al., 2017) [94]. Despite solid experimental evidence human studies are less convincing: some epidemiological data link increasing latitude and consequent decrease sunlight exposure with higher prevalence of multiple sclerosis [95,96,97], type I diabetes [98,99,100], and IBD [101]. It is clear that such differences may be due to genetic and lifestyle factors other than 25(OH)D_3_ levels. Other epidemiological data reinforcing the hypothesis of a link between sun exposure, vitamin D synthesis, and the risk of developing multiple sclerosis stem from the observation that subjects born in months associated with lower 25(OH)D_3_ level in the northern hemisphere (April) are at higher risk of developing the disease, whereas patients born in October (higher vitamin D levels) are at lower risk [102].

Some studies correlated vitamin D dietary intake and the prevalence of autoimmune diseases as rheumatoid arthritis [103] and type 1 diabetes mellitus [104,105], however, the correct evaluation of vitamin D intake is challenging as it is based on patient recall. To bypass the challenging measurement of vitamin D intake and sun exposure, levels of 25(OH)D_3_ in the serum can be useful, and, indeed, low levels of 25(OH)D_3_ in the serum of patients affected by autoimmune diseases with respect to healthy controls have been found [106,107,108,109,110,111,112]. Nevertheless, these studies demonstrated a correlation and not a causal relationship. 

Intervention studies with different doses of vitamin D_3_ in autoimmune diseases lead to different outcomes, recently we demonstrated that a bolus of vitamin D_3_ (300,000 UI) in patients affected by early rheumatoid arthritis is effective in ameliorating general health, however, we found no effect on disease activity nor on inflammatory markers and T cells subset [73]. In patients affected by type 1 diabetes clinical intervention studies with vitamin D_3_ or hydroxylated analogs have been disappointing, as no clinical study has demonstratedan effect of vitamin D in ameliorating glucose metabolism and insulin secretion [113,114], however, in a small prospective trial in children with type 1 diabetes autoantibodies 1,25(OH)_2_D_3_ administration decreased the serum glutamic acid decarboxylase 65 (GAD65) autoantibody, pointing to some immunomodulation of 1,25(OH)_2_D_3_ [115]. 

In addition to autoimmune diseases vitamin D has also been implicated in the control of other inflammatory conditions, such as cardiovascular diseases: in animal models vitamin D_3_ administration reduces macrophage production of pro-inflammatory cytokines, and decreases atherosclerosis and inflammation in the epicardial adipose tissue [116,117]. In humans an association between low 25(OH)D_3_ level and increased activation of inflammatory pathway in epicardial adipose tissuein patients affected by coronary artery disease has been described [118]. Vitamin D deficiency has been linked to cardiovascular disease not only by the modulation of inflammatory pathways, but also through the modulation of endothelial function, the effect on arterial stiffness, and a possible beneficial role on atherosclerotic plaque formation. However, this topic is beyond the scope of this review. For further insight in the role of vitamin D in the pathogenesis of cardiovascular disease see the review by Apostolakis and coll. [119].

## 8. Conclusions

In summary, several studies point to an important role of vitamin D as an immunomodulator, and strong data demonstrate a role for 1,25(OH)_2_D_3_ in increasing the ability of the innate immune system to fight against pathogens, whereas data on the effect of 1,25(OH)_2_D_3_in the modulation of acquired immune system are more controversial. There is no general consensus on the desired level of 25(OH)D_3_ to achieve immunomodulatory effects, thus, there is no current indication for vitamin D_3_ supplementation in patients with infections and/or autoimmune diseases. Further studies are needed to clarify the role of vitamin D as immunomodulator in humans.
